# Calcium-Binding Proteins as Determinants of Central Nervous System Neuronal Vulnerability to Disease

**DOI:** 10.3390/ijms20092146

**Published:** 2019-04-30

**Authors:** Richard Fairless, Sarah K. Williams, Ricarda Diem

**Affiliations:** 1 Department of Neurology, University Clinic Heidelberg, 69120 Heidelberg, Germany; S.Williams@dkfz-heidelberg.de (S.K.W.); Ricarda.Diem@med.uni-heidelberg.de (R.D.); 2 Clinical Cooperation Unit (CCU) Neurooncology, German Cancer Consortium (DKTK), German Cancer Research Center (DFKZ), 69120 Heidelberg, Germany

**Keywords:** calcium-binding protein, calbindin, calretinin, parvalbumin, neurodegeneration, vulnerability

## Abstract

Neuronal subpopulations display differential vulnerabilities to disease, but the factors that determine their susceptibility are poorly understood. Toxic increases in intracellular calcium are a key factor in several neurodegenerative processes, with calcium-binding proteins providing an important first line of defense through their ability to buffer incoming calcium, allowing the neuron to quickly achieve homeostasis. Since neurons expressing different calcium-binding proteins have been reported to be differentially susceptible to degeneration, it can be hypothesized that rather than just serving as markers of different neuronal subpopulations, they might actually be a key determinant of survival. In this review, we will summarize some of the evidence that expression of the EF-hand calcium-binding proteins, calbindin, calretinin and parvalbumin, may influence the susceptibility of distinct neuronal subpopulations to disease processes.

## 1. Introduction

Calcium, in addition to its essential role as a mediator of intracellular signaling, also serves as a key juncture in the process of neurodegeneration [1]. Thus, multiple injury pathways converge to induce an excessive rise in intracellular calcium levels which in turn activate a cascade of proteolytic enzymes, such as calpains and caspases, resulting in the onset of apoptosis. Therefore, the maintenance of calcium homeostasis within neurons is essential to their well-being, involving several mechanisms. These include: extrusion of calcium across the plasma membrane through gradient-driven calcium-permeable channels (e.g., the sodium-calcium exchanger) and active transfer via pumps (e.g., the plasma membrane calcium ATPase); uptake into intracellular stores such as the mitochondria or endoplasmic reticulum; or through binding to intracellular calcium-binding proteins (CaBPs).

Many different CaBPs exist with the largest family, the EF-hand CaBPs, consisting of over 240 currently identified members [2]. EF-hand proteins consist of one or more EF-hand domains composed of a highly conserved sequence of 12 amino acids which can chelate a single Ca^2+^ ion, flanked by two α-helices. Several EF-hand CaBPs are expressed ubiquitously, such as calmodulin, whereas others are differentially expressed in distinct neuronal populations. For example, hippocalcin is predominantly expressed by pyramidal cells of the hippocampus [3], whereas secretagogin is expressed by, amongst others, olfactory bulb neurons, granular layer interneurons [4] as well as amacrine cells and rod photoreceptors of the retina [5]. More recently, CaBPs such as caldendrin, expressed in the cerebral cortex, hippocampus and cerebellum [6,7], and calcium-binding protein 1, expressed in the cerebral cortex, hippocampus as well as cone bipolar and amacrine cells of the retina [7,8], have been discovered.

For the purposes of this review, we will focus on three well-known members of the EF-hand CaBP family, parvalbumin, calbindin D-28k (referred to as calbindin throughout this review) and calretinin. These CaBPs are abundantly expressed through-out the central nervous system (CNS), and have been extensively studied due to their varying distributions, thus serving as markers of discrete neuronal subpopulations. These CaBPs consist of multiple EF-hand domains, with parvalbumin containing three, and both calbindin and calretinin consisting of six domains, binding three, four, and five Ca^2+^ ions, respectively [9].

All three of these CaBPs have a high-binding capacity for calcium, although their kinetics appear to differ, for example parvalbumin is reported to exhibit slow-binding kinetics [2,10]. Due to their differential neuronal distribution and also the varying susceptibilities of differing neuronal populations to degeneration under various disease conditions, we will review here the relationship between the CaBP expression profile of neuronal populations and their susceptibility to neurodegeneration. Since neurodegeneration is known to affect specific neuronal subpopulations differently in an array of neurological diseases [11,12,13,14], and that the mechanisms underlying this are not fully understood [15], increasing our understanding of these processes will hopefully aid the development of effective neuroprotective strategies for the future.

## 2. CNS Distribution and Physiological Function of Neuronal CaBPs

**Figure 1:**
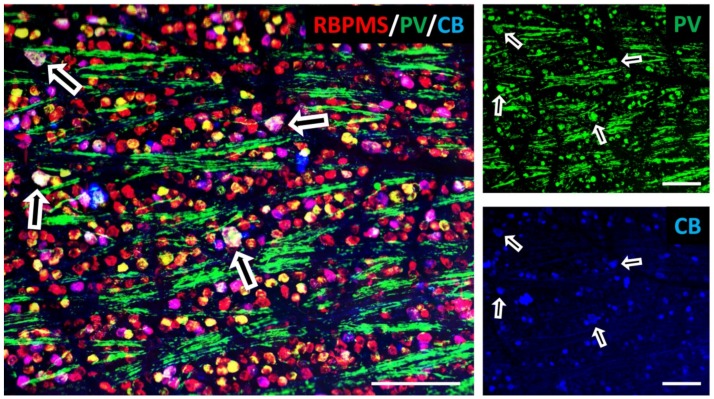
Retinal whole-mount from a Brown Norway rat labelled with antibodies against RNA-binding protein with multiple splicing (RBPMS, red; #ABN1376, Millipore, Darmstadt, Germany; 1:250) to identify retinal ganglion cells; and the calcium-binding proteins parvalbumin (PV, green; #P3088, Sigma-Aldrich, St. Louis, MO, USA; 1:200) and calbindin (CB, blue; #300, Swant Inc., Marly, Switzerland; 1:200). Individual color channels are shown as indicated. White arrows indicate retinal ganglion cells expressing both parvalbumin and calbindin. Scale bar = 100 µm. All experiments involving animal use were performed in compliance with the relevant laws and institutional guidelines of Baden-Württemberg, Germany. Previously unpublished data (R.F.).

The CaBPs, parvalbumin, calbindin, and calretinin, have been studied for several decades now, receiving particular focus due to their differential expression across the CNS. Many subpopulations of neurons have been reported to express one or more of these CaBPs (Table 1), and those mentioned here are in no way an exhaustive list. For example, many GABAergic interneurons have been reported to express parvalbumin, such as basket cells of the cortex and hippocampus [16], amacrine subpopulations in the retina [17,18,19], Purkinje cells of the cerebellum [20], and also interneurons of the cortex [21]. In addition, glutamatergic neurons, such as subpopulations of retinal ganglion cells [22] and corticostriatal projection neurons [23], also express parvalbumin. Calbindin-expressing neurons are similarly widely distributed and include cerebellar Purkinje cells [24,25], various hippocampal subpopulations including granule cells of the dentate gyrus [24] and superficial CA1 pyramidal neurons [26], as well as cortical populations [27]. Calretinin was first discovered in the retina (after which it was named), where it is expressed by subsets of retinal ganglion cells, amacrine and horizontal cells [28]. In addition, it is found in interneurons of the cortex [29] and hippocampus [30], as well as cerebellar granule cells [31]. Although most neurons appear to express either one or other of the CaBPs, some populations also express more than one [32] including multipolar cells of the somatosensory cortex [27], rat spinal and cranial sensory ganglia [33], non-GABAergic neurons of the rat spinal cord [34], and also some retinal ganglion cells (see Figure 1).

Elucidation of the physiological function of CaBPs is the focus of on-going research. Neurons require the ability to efficiently buffer calcium since active extrusion of calcium places a high metabolic demand on the cell, and great amounts of energy are already needed to reestablish electrochemical gradients after action potentials and synaptic release [47]. In addition to their roles as intracellular calcium buffers, or more specifically, ‘through’ their roles as intracellular calcium buffers, it is now becoming clear that CaBPs also help shape the synaptic responses and cellular excitability of neurons. Thus, their different kinetic and buffering properties might be suited to the specific requirements of the neuronal populations which express them. All three are classified as high-capacity calcium-binding proteins, but differences in these capacities are observed, with parvalbumin reported to have a dissociation constant (Kd) of 9 nM (in the absence of magnesium), calbindin of 393 nM, and calretinin of 1.5 µM [10]. The higher calcium-binding capacity of parvalbumin might be reflected in the strong correlation reported between parvalbumin expression and the fast-firing properties of hippocampal interneurons [48]. Conversely, due to the more limited calcium-buffering capacity of calretinin it has been suggested that, unlike parvalbumin and calbindin, it should be considered a ‘calcium modulator’ rather than a classical calcium buffer [49]. Furthermore, the kinetics of calcium binding varies between CaBPs; for example, parvalbumin displays much slower binding kinetics, possibly reflecting the co-affinity of its calcium-binding sites for magnesium, with Mg^2+^ ions needing to be displaced before calcium binding can occur [2]. In addition, calretinin displays cooperativity in calcium binding between the different EF-hand domains, with an increasing calcium-binding affinity as the calcium concentration increases [2].

Through the use of knockout mice, the influence of CaBPs on neuronal function has been further elaborated. Deletion of either parvalbumin, calbindin or calretinin has been shown to have important consequences for synaptic plasticity. Specifically, parvalbumin ablation slowed the decay of presynaptic calcium signals within the calyx of Held, and also the decay in paired-pulse facilitation [50]. Similarly, calbindin-deficient mice had altered paired-pulse facilitation [51] and long term potentiation [52] within hippocampal slices. Furthermore, calretinin knockouts had impaired long-term potentiation within the dentate gyrus [53]. These effects reflect the ability of CaBPs to modify the spatial and temporal properties of calcium transients occurring during synaptic activity.

Collectively, CaBPs appear to have different functional properties which may be suited to the excitability and synaptic characteristics of the neuronal population in which they are expressed. However, due to their different kinetics and buffering capacities for calcium, as well as their different expression levels within the cell, this may also have implications for the cell’s ability to cope with toxic elevations in intracellular calcium.

## 3. CaBPs as Markers for Neuronal Vulnerability to Disease?

It has been observed for several decades now that neuronal populations expressing particular CaBPs may be more vulnerable to, or conversely resistant to, neurodegeneration. This has predominantly been inferred through descriptive studies, where the CaBP expression profile primarily serves as a marker to differentiate neuronal populations, but claims of a more causal relationship beyond mere correlation have also been made. However, a closer look at the literature reveals that all three CaBPs discussed in this review are represented amongst both vulnerable and invulnerable neuronal populations in different regions of the CNS and as a result of a wide-range of diseases and disease models (summarized in Table 2).

Generalizations are hard to make and causality is even harder to determine from such correlative studies. However, there does appear to be an emerging consensus—namely, that parvalbumin-positive neurons (particularly interneuronal populations) are more susceptible to degeneration. In contrast, calbindin- and calretinin-positivity is more closely associated with resistance. This is further supported by the observation that immature granule cells of the dentate gyrus do not express calbindin and are equally vulnerable to ischemic injury as other neuronal populations. However, upon maturation they simultaneously start expressing high levels of calbindin whilst becoming resistant to ischemia [62].

A more conclusive methodology to determine any causal relationship between the CaBP expression profile of a given neuronal population and their susceptibility to neurodegeneration has been through the use of gene manipulation. In this manner, both overexpression and knockdown of a given CaBP can be achieved both in vivo and in vitro, and its influence on the fate of the neurons in question can be determined in a more precise manner than a correlative analysis (Table 3). This is particularly so due to the potential to analyze the contribution of a given CaBP in a defined population whilst avoiding a myriad of other major and subtle discrepancies which define the different neuronal populations.

Many studies report a correlation between CaBP expression and neuronal survival in agreement with the in vivo reports, but there are also many studies demonstrating a lack of influence, or even the opposite effect than that reported in the disease setting. For example, although most studies listed in Table 3 demonstrate that overexpression of calbindin has neuroprotective effects [73,74,78,79,80], in most cases its knockdown had no influence on neuronal survival [51,72,76]. Similarly for calretinin, some studies demonstrated overexpression to be neuroprotective [73,74] whereas another study showed a lack of effect [81]. In this case, Kuźnicki et al. [81] exposed a neuronal cell line to a different insult which will be discussed further in the next section. Upon knockdown of calretinin, however, no influence on neuronal survival was reported in response to kainic acid injection into the hippocampus [72]. For parvalbumin, interpretation of data is a bit more complicated due to the proposed susceptibility of parvalbumin-expressing neurons in vivo. In this context, parvalbumin may not be protective when expressed physiologically either due to its physical properties (kinetics and binding affinities), or, alternatively, due to the low level of expression. Therefore, studies demonstrating over-expression to be either protective [71] or to have no influence [73,74] could both be reconciled with the in vivo setting. Similarly, its knockdown had no effect on neuronal survival [72], again in keeping with a lack of protection.

To reconcile these different reports, it must be first noted that the experiments have all focused on different neuronal populations and insults, whereby the different CaBPs could have very different roles under these conditions. In addition, there may be a discrepancy between the in vivo and in vitro studies relating to the severity of insult. Application of an exogenous neurotoxin may inflict greater injury than more subtle changes in the cellular environment experienced during disease (a case-in-point would be application of NMDA to simulate NMDA receptor activation by glutamate, an excitatory amino acid that unlike its analogue can be cleared from the extracellular milieu by glutamate transporters). In addition, the duration or severity of the challenge is also important, with CaBPs being proposed only to efficiently protect against short, moderate excitotoxic insults [82], as demonstrated by the ability of calbindin and calretinin to protect against limited (2 h) but not prolonged NMDA exposure [74]. This may explain discrepancies arising due to distances from the primary insult. For example, Figueredo-Cardenas et al. [82] reported that calbindin-expressing neurons had enhanced survival compared to neurons expressing parvalbumin at the lesion edge following quinolinic acid injection, but no differences within the lesion itself. Similarly, investigation of neuronal survival to blood glutamate in the area postrema found calretinin-expressing neurons to be differentially sensitive depending upon their position, presumably reflecting the variable capillary permeability in different regions of this circumventricular organ [83]. A second difference between the in vivo and in vitro investigations may involve an increased susceptibility of neurons to injury following their removal from their in vivo setting, whereby a loss of endogenous supplies (such as glia-secreted factors) and a disturbance in their network connectivity may result in a loss of neuroprotective support [84,85]. Thus, these types of experiments may not accurately reflect the complexity of the in vivo disease paradigm.

An obvious explanation for the difference in the knockout studies would be the potential for compensation for CaBP ablation through the upregulation of other family members. However, following knockdown of calbindin, Airaksinen et al. [76] reported a lack of calretinin upregulation in the surviving neurons in an MPTP-induced model of Parkinson’s disease. In addition, a global investigation was performed by Schmidt et al. [86] demonstrating that following ablation of parvalbumin or calbindin expression, no upregulation of any other CaBPs were detectable within cerebellum lysates by ^45^Ca^2+^-overlay blotting. However, compensation may be achieved through alternative means, for example, in response to parvalbumin ablation, it has been reported that mitochondrial volume increases [20]. Similarly, overexpression of parvalbumin results in a decrease in mitochondrial density [87] demonstrating that there exists a crosstalk between CaBPs and mitochondria which may serve to balance the calcium homeostatic machinery.

Thus, there are many examples of studies both supporting and opposing the concept that CaBP expression and resistance to calcium-mediated injuries are linked. This may reflect the fact that there appear to be more factors in addition to CaBP expression that determine neuronal susceptibility to degenerative processes, such as the mode of injury and other subgroup-specific factors. Neurons are highly specialized cells, explaining the numerous distinctive cell populations within every region of the CNS, and the CaBP expression profile may just reflect one tailored facet of their function. Other important aspects of neuronal biology which may affect the ability of CaBPs to provide protection against insults include both their localization within the cell, and the regulation of their expression.

## 4. Subcellular Localization of CaBPs

CaBPs are essentially soluble cytosolic calcium buffers, meaning that they can freely distribute themselves within the cell. Thus, due to the presence of multiple immobile calcium buffers within the cell which limit the spatio-temporal longevity of calcium signals, soluble buffers can increase the range over which calcium can be distributed [88]. Differences in CaBP mobility, for example between spines (where synaptic calcium influxes occur) and the parental dendrites, exist—for example, calbindin diffusion was reported to be ~2 fold higher than that for parvalbumin [10]. This difference in diffusion kinetics may explain why in Purkinje cells, calbindin, more than parvalbumin, was found to transport the major fraction of calcium from the spine to the dendrite [86]. The impact of this on neurodegeneration is not clear, but it may explain the observation that overexpression of parvalbumin in neocortical neurons increased their susceptibility to NMDA-induced excitotoxicity [70]. It is conceivable that CaBP-mediated redistribution of calcium contributes to an uncoupling of synaptic calcium signals from microdomains where neuroprotective signaling has been reported to be elicited, concomitantly increasing the non-synaptic calcium pool associated with activation of neurodegenerative pathways [89].

The subcellular localization of different CaBPs also appears to vary, which is most clearly demonstrated in neurons coexpressing multiple CaBPs. For example, in retinal neurons coexpressing calbindin, calretinin and parvalbumin, these CaBPs were compartmentalized within various different neuronal structures [5]. Calretinin has been reported to colocalize with the NMDA receptor subunit 1 (NR1) [90], perhaps explaining the report that calretinin is actively relocated from the cytosol to the submembranous region during the development of nucleus magnocellularis cells [91]. This might also explain the different CaBP influences on neuronal survival contingent upon the mode of calcium entry into the cell [71]. For example, calretinin-expressing hippocampal neurons were more resistant to glutamate receptor activation than to calcium elevation mediated by an ionophore [92]. Similarly, Kuznicki et al. [81] reported a lack of calretinin-mediated protection in PC12 cells in response to ionophore application. In this manner, ionophore-derived calcium might not be entering the cell in the vicinity of a CaBP microdomain, thereby possibly bypassing its major buffering effect.

## 5. Regulation of CaBP Expression

An important distinction should be made between the downregulation in expression of a particular CaBP, and the loss of the neuron altogether, as described in Table 2. Thus, where a relative decrease in a discreet neuronal population expressing a particular CaBP is observed, concluding that these cells are more vulnerable might be too simplistic, ignoring the potential for an alteration in the CaBP expression profile. This might reflect a protective response of the neuron against neurodegeneration that arises during pathophysiological conditions. Potentially neuroprotective increases in CaBP expression have been reported following acute injuries such as axotomy [93,94] or head trauma [95], and also in response to glutamate receptor activation [96,97] or exposure to other neurotoxins such as MPTP [98]. In addition, olfactory bulbectomy was shown to result in concomitant increases and decreases in different CaBPs [99]. Thus, it could be argued that if a disease or insult does not result in neuronal death, the neuron has the capacity to change CaBP expression as part of a protective response.

The influence that CaBP regulation may play on cellular survival has been most extensively addressed in the context of aging—where an age-related decrease in calbindin expression has been reported in several regions including the basal forebrain [60,100], cerebellum, corpus striatum and brainstem [101], and sympathetic neurons of the pelvic ganglion [102] and hippocampus [103]. In addition, this downregulation appears to be specific to calbindin, and was not observed for parvalbumin or calretinin in the perirhinal cortex [104] or hippocampus [103]. Within the perirhinal cortex, this decrease in calbindin immunoreactivity appears to be as a result of a downregulation in expression rather than selective neuronal loss since the neuronal density was not seen to significantly decrease [105]. This downregulation associated with aging might not only render neurons more susceptible to cell damage and death [101], but has also been postulated to increase vulnerability to subsequent degeneration in association with Alzheimer’s disease [60,77]. The transcription factor ΔFosB has been demonstrated to mediate a decrease in calbindin expression following seizures in mouse models of epilepsy and Alzheimer’s disease [106], and thus may be involved in age-related downregulation of calbindin. Precisely why calbindin is downregulated during the aging process is not yet understood, but may be associated with several cognitive [101,106] and metabolic [75] changes that occur during aging.

A further example of CaBP regulation is through the action of brain-derived neurotropic factor (BDNF). Exposure of cortical [107] and hippocampal [108] neurons to BDNF has been shown to induce expression of calbindin, which may be mediated through the transcription factor c-Fos. In addition, a concomitant decrease in calretinin expression was also reported following treatment with BDNF [109]. Due to the well-characterized neuroprotective effects of BDNF [110,111,112], it is intriguing to postulate that the resultant change in the CaBP expression profile allows the neuron to adopt a protective stance. Further research into the regulatory aspects of CaBP expression will hopefully help us to understand the processes involved and how these can be manipulated in the development of neuroprotective therapies.

## 6. Conclusions

There have been many studies highlighting the association between neuronal susceptibly to degenerative processes and the expression of different CaBPs. However, upon further investigation, the causal relationship between the two is clearly much more complex. Firstly, the influence of CaBP expression on neuronal vulnerability appears to depend heavily upon the disease or disease model, where the severity of the injury, and also the precise pathway of calcium elevation, may be crucial. Secondly, the neuronal population involved is also an important factor, with CaBP expression profiles appearing to be finely tuned to meet the specific functional requirements of the cell type. There are also numerous other factors involved in determining the susceptibility of particular neuronal populations to degeneration including, but not limited to, their circuit connectivity, trophic support network, relative contributions of synaptic to extrasynaptic NMDA receptors, and also their energy requirements. With respect to the latter, it is of note that the presence of particular CaBPs may reflect the activity of the neurons in which they are expressed. For example, high CaBP expression has been associated with high rates of calcium influx and/or intracellular release. However, these same neurons might also be those most at risk of degeneration due to the same high energy demands placed upon them.

Recent experiments have led to an emerging perspective that some CaBPs, in addition to their roles as calcium buffers, may also function as calcium sensors. Thus, both calbindin [10] and calretinin [113] have been reported to display properties suitable for functioning as a sensor, namely substantial conformational rearrangement upon calcium binding and potential interaction with proteins involved in calcium signaling. So far, this has been mainly investigated in vitro or in non-neuronal cells, but it does open up the possibility for therapeutic targeting of undesirable signaling pathways, as is being explored for other CaBPs such as calmodulin [114].

Finally, it is becoming increasingly clear that CaBP expression profiles are dynamic during development, aging and following disease or injury. Further understanding of factors controlling CaBP expression and how neurons regulate this under pathophysiological conditions may allow neuroprotective strategies to be developed, tailored to the specific neuronal populations at risk.

## Figures and Tables

**Table 1:** Summary of selected CNS neuronal populations and their CaBP expression.

CaBP	CNS Distribution	Reference
**Parvalbumin**	Amacrine cells	[17,18,19]
	Cerebellar Purkinje neurons	[20,35]
	Cortical basket cells	[16,36]
	Cortical interneurons	[21,37]
	Corticostriatal projection neurons	[23]
	Hippocampal interneurons	[16,38]
	Retinal ganglion cells	[22,39]
**Calbindin**	Cerebellar Purkinje neurons	[24,25]
	Cortical nonpyramidal neurons	[27,40]
	Granule cells of the dentate gyrus	[24,41,42]
	Hippocampal pyramidal neurons	[26,42]
	Retinal ganglion cells	[39]
**Calretinin**	Amacrine cells	[28,43]
	Cerebellar granule cells	[31,44]
	Cortical interneurons	[29]
	Hippocampal interneurons	[30,45]
	Retinal ganglion cells	[28,46]

**Table 2:** In vivo correlation of CaBP expression and susceptibilities of neuronal populations to disease and disease models.

CaBP	Resistant (+) or Susceptible (−) to Neurodegeneration?	Neuronal Population/Region	Disease/Model	Ref.
**Parvalbumin**	**+**	Hippocampus CA1 region	Experimental ischemia	[54]
	**−**	Ganglion cell layer of retina	Retinal ischemia	[55]
	**−**	Motor cortex	Multiple sclerosis	[21]
	**−**	Hippocampus	Kainic acid injection	[56]
	**−**	Hippocampus	Kainic acid injection of aged mice	[57]
	**−**	Entorhinal cortex	Alzheimer’s disease	[58]
	**−**	Hippocampal GABAergic interneurons	Schizophrenia	[59]
**Calbindin**	**+**	Ganglion cell layer of retina	Retinal ischemia	[55]
	**+**	Cholinergic neurons of basal forebrain	Alzheimer’s disease	[60]
	**+**	Midbrain dopaminergic neurons	Models of Parkinson’s disease	[61]
	**+**	Dentate granule cells	Experimental ischemia	[62]
	**−**	Pyramidal cells of CA1 hippocampus	Experimental epilepsy	[63] ^1^
**Calretinin**	**+**	Substantia nigra neurons	Parkinson’s disease	[64]
	**+**	Cerebral cortex	Aging	[65]
	**+**	Hippocampus	Epilepsy	[66]
	**+**	Striatum	Huntington’s disease	[67]
	**+**	Dopaminergic neuronal subpopulations	Parkinson’s disease	[68]
	**+**	Substantia nigra neurons	Parkinson’s disease	[69]

^1^ Freund et al., 1992 give numerous examples of CaBP expression and neuronal populations with positive and negative correlations to vulnerability.

**Table 3:** CaBP modulation through overexpression or knockout under in vivo and in vitro conditions.

CaBP	Conditions	Gene Manipulation (↑, Overexpression; ↓, Knockout)	Neuronal Population	Insult	Supportive of Neuroprotective Role?	Ref.
**PV**	In vitro	↑	Cortical neurons	NMDA exposure	No	[70]
	In vivo	↑	Spinal motor neurons	KA exposure	Yes	[71]
	In vitro	↓	Temporal lobe	Epilepsy model	No	[72]
	In vitro	↑	Neuroblastoma-retina hybrid cells	Glutamate exposure	No	[73]
	In vitro	↑	P19 cell line	NMDA exposure	No	[74]
**CB**	In vivo	↓	Hippocampus	Age-mediated decline	Yes	[75]
	In vivo	↓	Midbrain dopaminergic neurons	MPTP injection	No	[76]
	In vivo	↓	Subiculum	Alzheimer genetic model	Yes	[77]
	In vivo	↓	Hippocampal CA1 pyramidal neurons	Ischemia model	No	[51]
	In vitro	↑	SOD-1 mutant motor neurons	Glutamate exposure	Yes	[78]
	In vitro	↑	Hippocampal neurons	Glutamate exposure	Yes	[79]
	In vivo	↑	Hippocampus	KA and 3-AP exposure	Yes	[80]
	In vitro	↓	Temporal lobe	Epilepsy model	No	[72]
	In vitro	↑	Neuroblastoma-retina hybrid cells	Glutamate exposure	Yes	[73]
	In vitro	↑	P19 cell line	NMDA exposure	Yes	[74]
**CR**	In vitro	↑	PC12 cell line	Ionophore exposure and serum/growth factor withdrawal	No	[81]
	In vivo	↓	Temporal lobe	Epilepsy model	No	[72]
	In vitro	↑	Neuroblastoma-retina hybrid cells	Glutamate exposure	Yes	[73]
	In vitro	↑	P19 cell line	NMDA exposure	Yes	[74]

3-AP, 3-acetylpyridine; CB, calbindin, CR, calretinin; KA, kainic acid; MPTP, 1-methyl-4-phenyl-1,2,3,6-tetrahydropyridine; NMDA, N-Methyl-D-aspartate; SOD-1, superoxide dismutase-1; PV, parvalbumin.
